# Tobacco Vendors’ Perceptions and Compliance with Tobacco Control Laws in Nigeria

**DOI:** 10.3390/ijerph20227054

**Published:** 2023-11-12

**Authors:** Omotayo F. Fagbule, Catherine O. Egbe, Olalekan A. Ayo-Yusuf

**Affiliations:** 1 Department of Public Health, Sefako Makgatho Health Sciences University, Ga-Rankuwa, Pretoria 0208, South Africa; catherine.egbe@mrc.ac.za; 2 Department of Periodontology and Community Dentistry, Faculty of Dentistry, College of Medicine, University of Ibadan, Ibadan 200212, Nigeria; 3 Mental Health, Alcohol, Substance Use and Tobacco Research Unit, South African Medical Research Council, Pretoria 0001, South Africa; 4 Africa Centre for Tobacco Industry Monitoring and Policy Research (ATIM), School of Health Systems and Public Health, University of Pretoria, Pretoria 0002, South Africa; lekan.ayo-yusuf@up.ac.za

**Keywords:** retailers, cigarette sellers, tobacco control legislation, tobacco control policies, WHO FCTC, low and middle-income countries, tobacco vendors

## Abstract

Tobacco vendors are critical stakeholders in the tobacco supply chain. This study examined their perception, compliance, and potential economic impact of Nigeria’s tobacco control laws related to the retail setting. This was a qualitative study involving in-depth interviews of 24 purposively selected tobacco vendors. The face-to-face interviews were aided by a semi-structured interview guide, audio-recorded, transcribed verbatim, and analyzed using thematic analysis with NVivo version 12. Five themes emerged, encompassing reasons for selling tobacco, awareness, perception, compliance with tobacco sales laws, the potential economic impact of the laws, and law enforcement activities. Vendors commenced tobacco sales due to consumers’ demand, profit motives, and advice from close family relatives. They were unaware and non-compliant with most of the retail-related laws. Most participants had positive perceptions about the ban on sales to and by minors, were indifferent about the ban on Tobacco Advertising Promotion and Sponsorships (TAPS) and product display, and had negative perceptions about the ban on sales of single sticks. Most vendors stated quitting tobacco sales would not have a serious economic impact on their business. In conclusion, the vendors demonstrated limited awareness and non-compliance with various retail-oriented tobacco control laws in Nigeria. Addressing these gaps requires targeted educational campaigns and effective law enforcement strategies to enhance vendors’ compliance.

## 1. Introduction

Tobacco use is estimated to kill about 8 million people globally every year, and 80% of these deaths occur in low- and middle-income countries (LMICs), which includes Nigeria [1]. These deaths are a result of several diseases associated with tobacco use [2]. A major vector of the causative agent, tobacco products, is the tobacco industry and its front groups and allies [3]. The tobacco industry aggressively recruits new customers as replacements for dying smokers and those who quit [4]. 

Tobacco vendors/retailers are critical stakeholders in the tobacco supply chain [5]; they are responsible for placing tobacco products into the hands of the users. The activities of tobacco vendors are important areas targeted by the tobacco industry and tobacco control laws [6,7]. Tobacco control laws, including the ban on sales to and by minors, the ban on tobacco advertising, promotion, and sponsorships (TAPS), and the ban on product displays, are proven methods of reduce ng the prevalence of tobacco use [8,9,10,11]. Hence, Section 15 (1) of Nigeria’s National Tobacco Control Act, 2015 (NTCA) prohibits the sale of tobacco and tobacco products to and by a minor (below 18 years). Section 15 (3) also obligates a tobacco vendor to display signage stating that tobacco sales to minors are prohibited, and Section 15 (5) prohibits sales of single cigarette sticks. Similarly, Section 12 (1), (2) (b), and the First Schedule (3) ban TAPS, including product display, with the exception being consenting adults [12]. However, despite these tobacco control laws in Nigeria [12], the prevalence of tobacco use continues to increase in the country, especially among children and adolescents [13]. This suggests that there may be problems with the implementation or compliance with the laws. However, there has been a lack of empirical data to confirm or refute these possible reasons. 

One study carried out in the Ibarapa community of Oyo State, Nigeria, reported that about two-thirds of the vendors considered tobacco sales profitable, but the majority were willing to participate in tobacco control programs [7]. However, there are gaps this study did not address. Firstly, the study did not report the vendors’ awareness, perception, and compliance with the existing laws. Secondly, the study was conducted in a rural setting, and considering the differences in the socio-demographics of the populace in rural and urban areas in Nigeria, it is important to know what vendors in urban areas think about tobacco control laws. Hence, this study was conducted to provide insight into the perception of tobacco vendors in urban and peri-urban areas and the extent of their compliance with the laws relating to tobacco retailing in Nigerian settings. The potential economic implications of stopping tobacco sales on the vendors were also explored.

## 2. Materials and Methods

### 2.1. Study Design

This study employed a cross-sectional design. It was a qualitative study that used in-depth interviews. Interviews were aided by a semi-structured guide (Supplementary Materials). 

### 2.2. Study Area and Population

The study was carried out among tobacco vendors in Ibadan, Oyo State, Nigeria. Ibadan is the biggest city in sub-Saharan Africa, has both urban and suburban areas, and is home to one of the biggest tobacco factories in West Africa, with a strong tobacco distribution network within the city [14]. Tobacco vendors are commonly found near educational institutions, commercial parks, markets, and adjoining streets of these places [14]. A sample of vendors selling on the streets and those who have kiosks/corner shops in and around educational institutions, commercial parks, and markets were recruited into the study.

### 2.3. Participants’ Eligibility and Selection

A purposive sampling technique was employed to recruit a total of 24 participants from a diverse group of tobacco vendors. There was a good representation of the vendors based on demographics and location of retail outlets (roadside, near schools, commercial motor parks, and markets). Participants were adults (18 years and above) who sold one or more tobacco products, either alone or with other non-tobacco products in Ibadan, Nigeria. After interviewing the 24th participant, there was no new information, suggesting that data saturation had been reached [15].

### 2.4. Ethical Considerations

Approval for this study was received from the Sefako Makgatho Health Sciences University’s Research Ethics Committee (SMUREC/H/90/2021: PG) and the University of Ibadan/University College Hospital Ethics Committee (UE/EC/21/0201). A detailed explanation of the study was provided to all the participants. They all agreed to participate in the study and subsequently signed the informed consent forms.

### 2.5. Data Collection

One of the authors (OFF), with a Master’s degree and previous experience in qualitative studies, and who was specifically trained for this study, conducted all the interviews between December 2021 and January 2022. The interviewer was assisted by a trained and experienced assistant. The interviews were face-to-face with each participant in a relaxed atmosphere, mostly at their sales location, but devoid of distractions. The interviews were guided by a validated semi-structured interview guide and audio recorded. The questions were carefully constructed to explore participants’ perception and compliance with the laws prohibiting tobacco sales to and by minors, sales of single cigarette sticks, point of sale (POS) advertisements and product display, smoking in their stores/kiosks (public places), and having signage stating that tobacco sales to minors are prohibited. The participants’ awareness and opinions about the laws, their enforcement, and their willingness to abide by them were also sought. The interviewer asked follow-up questions based on the responses from the participants. The average interview time was about 25 min, with the range being 15–35 min. The interviewer and research assistant also maintained an observation sheet to record information such as the presence of tobacco advertisements, tobacco product displays, and the display of signage indicating tobacco sales to minors are prohibited. 

Twenty interviews were conducted in the Yoruba language and three interviews were in the English language. The voice-recorded interviews were transcribed verbatim after each interview and those conducted in Yoruba were subsequently translated into the English language. This was done concurrently and iteratively with the data collection to explore emergent ideas further in subsequent interviews.

### 2.6. Data Analysis

The transcripts were checked for errors, after which they were imported into a qualitative data analysis software—NVivo version 12 where thematic analysis of the data was conducted. A codebook (Supplementary Materials) was developed using mainly the deductive approach. An initial set of codes, based on the interview guide, served as the foundation of the codebook. These codes were subsequently refined and expanded to incorporate emerging themes and subthemes throughout the process of data analysis. 

One of the authors (OFF) generated the initial codes, and after the coding process, a repeated pattern of the codes was systematically identified and grouped into potential themes. An initial thematic map was created, and the themes were sorted into the main themes and sub-themes. A co-author (COE) also independently analyzed the data from one transcript and generated themes which were subsequently compared to the initial themes by OFF and areas of discrepancies were resolved after discussing with the second co-author (OAA). The study participants’ sociodemographic characteristics (age, sex, tribe, educational status, study area, location, and types of POS) were summarized and reported as frequencies and proportions.

## 3. Results

### 3.1. Sociodemographic Characteristics and Tobacco Use Status

The participants’ ages ranged from 31 to 70 years with a mean (±SD) age of 47.6 (±11.8) years. Most were females (n = 20), belonged to the Yoruba tribe (n = 22), and 12 (50.0%) had secondary-level education as the highest level of education. The majority were in urban communities (n = 16) and sold their tobacco products in shops (n = 14). Most participants had never used tobacco (n = 21) (Table 1).

### 3.2. Themes and Sub-Themes

There were seven main themes derived from the data inductively (Table 2).

#### 3.2.1. Theme 1: Vendors’ Reasons for Commencing Tobacco Sales

The most common reason the vendors provided for starting to sell tobacco products was the high demand from their customers who drink alcohol. The vendors felt compelled to start selling cigarettes because when those who intend to buy alcoholic drinks request cigarettes and the vendors do not have them, the potential customers usually go to other stores where they can buy both. Thus, the vendors lose out on selling their alcoholic drinks.

P20: *It is part of the business I am doing because I sell alcohol, and when people come to buy alcohol, they commonly ask for cigarettes too. Some will not want to buy alcohol if they do not see cigarettes, so that is the major reason why I added cigarettes sale to the business.*

The second most common reason was the need to feed their family. They believed tobacco sales would be very lucrative because many people, especially youths, were smoking cigarettes. Though many of them recognized that tobacco was harmful, the need to make a living and earn a profit was a greater motivation for them to sell.

Finally, the influence of the vendor’s close family relative also played an important role in their decision to start selling tobacco.

P15: *My mom used to sell cigarettes too, so I decided to add cigarettes sale to my pepper-grinding business, along with selling dry gins [alcoholic drinks].*

#### 3.2.2. Theme 2: Vendors’ Awareness of the Tobacco Control Laws Related to the Retail Settings

Most of the participants (n = 21) were unaware of any tobacco control law, even after the interviewer probed for the individual laws. The remaining three participants were only aware of the law banning tobacco sales to minors.

Interviewer: *What about a law stating that you do not sell cigarettes to young people below 18 years?*

P23: *There is nothing like that. They send young children to buy it; their brother will send them, and their father will send them, so there is no law forbidding it.*

P14: *Yes, it is written on it, 18 years and above. Apart from that, there is no other law.*

#### 3.2.3. Theme 3: Vendors’ Perception of the Laws

After establishing the vendor’s awareness of the various laws, the interviewer informed them about all the laws, and what they entail, and subsequently sought their views about each of the laws. The participants’ perceptions of the laws could be categorized as negative, positive, and indifferent, and they differed depending on the individual laws. 

All responses to the ban on sales to and by minors were positive; the participants supported the bans.

P16: *It will be good if they enact a law banning tobacco sales to minors.*

However, the ban on the sale of single sticks attracted negative perceptions from the participants. The participants’ major concerns were their lack of “capital” to buy products in bulk (cartons) so that they could sell in packs, as most were only able to buy a few rolls of cigarette packs. Secondly, most of their customers could not afford to buy cigarette packs. Finally, participants mentioned that selling cigarettes as single sticks was more profitable than selling them in packs.

P12: *The single sticks are the most profitable to sell. Because if you sell a whole cigarette pack at once, you will only gain ₦100 ($0.22), but if you sell it in single sticks, you may make as much as ₦250 ($0.55) or ₦200 ($0.44).*

Many of the participants were indifferent to the ban on tobacco advertisements and the law obligating vendors to have signage stating that minors are not allowed to buy tobacco. The vendors opined that cigarettes sell fast and do not need to be advertised. They stated that potential buyers would always seek out where they are sold. Also, they believed selling alcoholic drinks was enough advertisement since it is a popular notion that whoever is selling alcoholic beverages, especially those in sachets, would be selling cigarettes too.

P20: *Everybody knows that wherever they sell beer, hot drinks, and other alcoholic drinks, they will surely sell cigarettes there. So cigarettes sale do not need to post a banner [does not need to be advertised].*

#### 3.2.4. Theme 4: Vendors’ Compliance with the Tobacco Laws

The vendors’ compliance with the existing point-of-sales-related tobacco control law was generally poor. None of the participants was compliant with the laws banning sales of single cigarette sticks and those obligating them to have signage stating tobacco sales to minors are prohibited (Table 3).

Half of the participants engaged minors, including their children and grandchildren, to sell cigarettes on their behalf. A participant got parental consent to employ a 14-year-old girl as her salesgirl.

P12: *A child can sell. I said that because, for example, this child [pointed to a small child] was brought for me from Ogbomoso [another city in Oyo State] to assist me in selling my goods [including cigarettes] when I am not in the shop. She is less than 15 years old because I specifically asked for a minor.*

Ten out of the 12 participants did not have minors selling for them because they had no children available. Only two participants said they would not allow their children to sell tobacco, and their reason was not that they knew about the law banning tobacco sales by minors but that they wanted their kids to concentrate on their studies.

Interviewer: *You don’t have any children who can help you sell?*

P17: *My children are still very young.*

Regarding the participants’ compliance with the ban on sales to minors, 18 (75%) of them were not compliant with the law. Although most of them claimed they do not sell cigarettes to minors, when probed further, it was discovered that what they meant was that they do not sell cigarettes to children who intend to smoke them. But they readily sold to minors who told them they were running errands for adults.

P15: *I sell to children, as long as they are not the ones smoking it, they can be sent to come and buy it from me, and I will sell it.*

When the participants were asked how they confirm the age of their customers who may be minors, they all responded that their judgment was usually based on the person’s facial maturity.

P18: *Once you see the person, you will know [if he/she is a minor], at least if you want to describe my age now, you’ll know how to describe it, so that’s how it is.*

Regarding their compliance with the ban on TAPS, most vendors did not have posters or stickers to advertise their tobacco products, although a few had these advertising aids. Similarly, about a third of the participants engaged in tobacco promotion by giving out free cigarettes to customers. They explained that sometimes the sales representatives of the tobacco industry give them free samples of their new products for their customers to try out. The aim was to provide new products, usually cheaper, as alternatives to the costlier tobacco brands.

P21: *I don’t advertise cigarettes; they come to ask themselves.*

P13: *When I go to Agbeni market [wholesalers], they might give me banners or table mats. The one I collected from “Yes” [cigarette brand] is there, I tied it around the counter for people to see it.*

P17: *Yes, those just manufacturing new cigarette brands might give us some [cigarettes]. Last month, some marketers came and gave us one pack to distribute to our customers to taste.*

**Figure 1:**
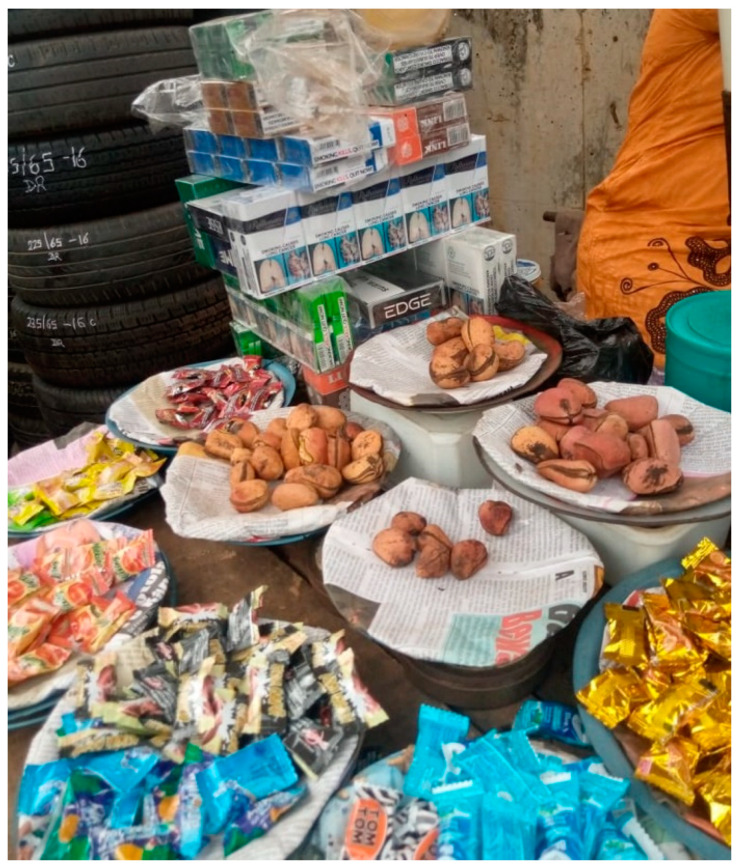
Picture showing rolls of cigarette packs carefully arranged for easy visibility and displayed alongside candies and other edibles in Ibadan, Nigeria, 2022.

**Figure 2:**
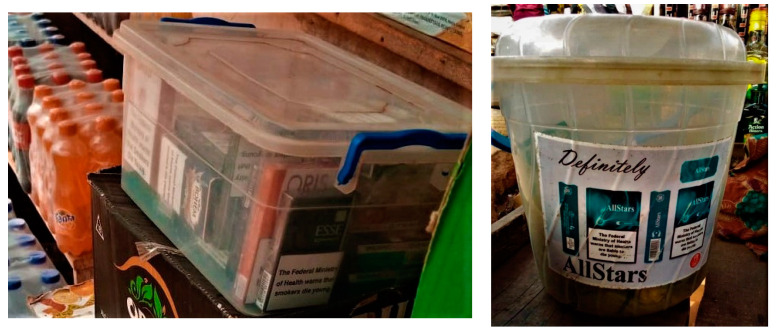
Pictures showing cigarette packs displayed in transparent buckets in Ibadan, Nigeria, 2022.

Based on the interviewer’s observation and responses from the vendors regarding product display, all the participants displayed tobacco products. These products (usually cigarette packs which are sometimes empty) were displayed where passers-by, including children, could easily see them. They were also often placed among non-tobacco products like biscuits, candies, and drinks (alcohol/non-alcohol) (Figure 1 and Figure 2).

Most of the vendors had transparent plasticware where they put the cigarette packs. Some of these plasticwares were branded by the tobacco companies and given to the vendors by the sales representatives. Some vendors also created special shelves where they arranged empty cigarette packs and placed the shelves in front of their shops to get the attention of potential buyers.

P12: *To make people know we sell it [cigarettes] here, we will display them outside. I displayed it outside, inside transparent plastic bowls/buckets.*

#### 3.2.5. Theme 5: Reasons for the Participants’ Compliance Status

Most participants were unaware of the tobacco control laws guiding tobacco sales in Nigeria, which was the main reason they were non-compliant with the laws. This reason was bolstered by the fact that many of them expressed their willingness to comply with most laws, except the ban on sales of single sticks, when the interviewer informed them. Although, some of the participants said they were willing to comply with the laws, they did not expect these laws to affect their business negatively.

P24: *I have never heard of any laws.*

P14: *Do we have any authority over the government? It is whatever regulation they bring that we will follow.*

Many of the participants stated that they usually do not sell tobacco to minors they believed wanted to use them because they were concerned about the potential health effects of tobacco use on children.

P17: *… That [addiction and health problems] is also the reason I don’t sell to children because what a child has been accustomed to from childhood will be very hard to leave later on.*

However, irrespective of their reservations about the health effects of smoking on children, some vendors still sold cigarettes to children they knew intended to smoke it. The most common reason the vendors sold cigarettes to minors who smoked was their focus on making profits.

#### 3.2.6. Theme 6: Potential Economic Effects of Stopping Tobacco Sales

The subthemes related to the potential economic impact of the tobacco business included “profit from tobacco sales”, “proportion of overall profit from tobacco sales”, and the “vendors’ perception of the potential economic impact of stopping tobacco sales”.

##### Profit from Tobacco Sales

The participants’ responses concerning the profit they make from tobacco sales can be divided into low-profit, high-profit, and high demand. Most (15) of the vendors reported that profit on the sales of tobacco products, especially cigarettes was meagre.

P14: *How much is the profit of cigarettes? It is not much. You will even see some that will come to smoke and ask to pay later, and you can’t fight them. When you sell a pack of cigarettes, maybe the profit you will make is ₦70 or ₦50 [$0.16 or $0.11].*

P20: *From morning until night, one might make a profit of ₦500 [$1.2] on cigarette sales.*

Other than the low profit margin on cigarette sales, some vendors complained that the customers usually do not want to pay. The customers often want the vendors to give them a few cigarette sticks as a “gift” for buying other non-tobacco products, especially alcohol.

P14: *There is not much profit from it. If you are not careful, you will sell a whole pack and will not make any profit. When people come and pick up cigarettes without paying immediately, you will not want to fight them so that they don’t see you as an angry person, but you may not see those debtors again.*

When the vendors were asked why they did not increase the prices to boost their profit, they explained that they find it challenging to raise cigarette prices unilaterally. This is because there are many tobacco vendors; thus, there is an increase in competition.

P16: *Yes, you cannot add [too much money] to the cigarettes prices. Customers will tell you they usually buy it cheaper somewhere else. And there are a lot of people selling cigarettes.*

In contrast to the low-profit margin, a few (3) participants reported a potential for a high-profit margin on cigarette sales, especially if the vendor has a huge capital to buy in cartons, similar to wholesalers.

P15: *When it comes to cigarettes, people buy them a lot from me. If I sell the different brands I have, sometimes, if God blesses my market, I make ₦1000 [$2.2] as profit daily. Like the one I am selling now, it’s a pack that remains like three sticks now, and I will at least sell more before night.*

Most vendors reported that many tobacco users visit their shops to buy cigarettes and may also buy other products. For some of them, the only reason they were still selling cigarettes, despite its low profit, was because it increased the traffic of potential customers visiting their shops/point of sales (POS).

P3: *I don’t profit from cigarettes sale, but it increases the number of customers who come to my shop.*

##### The Proportion of Overall Profit from Tobacco Sales

Most participants reported that the proportion of their overall profit from tobacco sales compared to other non-tobacco products that they also sell was small, while only one participant mentioned that it was about average (50%). Generally, the profit they made from selling other non-tobacco products was higher than what they made from cigarette sales.

Interviewer: *But let’s put it this way, if, at the end of today, you made ₦100 [$0.24] as profit from all the products (tobacco and non-tobacco) that you sold, how much out of that ₦100 would have come from tobacco/cigarettes sale?*

P22: *Maybe ₦20 [$0.05].*

##### Vendors’ Perception of the Potential Economic Effect of Ending Tobacco Sales on Their Overall Business

None of the participants believed that completely stopping tobacco/cigarette sales would seriously affect their overall business. In contrast, many said it would not affect their overall business, with a few stating they were already considering stopping tobacco sales. Others reckoned that it might cause a reduction in the number of people who patronize them, but it would not be enough to put them out of business altogether.

P9: *It won’t affect my overall profits because I have been thinking I want to stop selling it. This is because of how those smoking it behave. Some will even be commanding me, “go and find lighter or matches for us o”, you know, talking like touts, so I’m starting to lose interest in it.*

P3: *I won’t have to close down my business, but sales will be dull. I won’t have many customers visiting my shop.*

The vendors mentioned that they could sell other non-tobacco products instead of cigarettes.

P10: *If we are asked not to sell it [tobacco] again and pack up, I will go for other things. I used to cook Indomie and egg before and also drinks.*

#### 3.2.7. Theme 7: Vendors’ Experience with the Activities of the Enforcement Agents

The following sub-themes emerged during the discussion around the effectiveness of the laws:

##### Enforcement Agents Do Not Provide Education about the Laws

All participants mentioned that they have never received any information or education about Nigeria’s existing tobacco control laws, especially those related to the point of sale (POS).

##### Law Enforcement Agents Do Not Enforce Laws around Cigarettes/Tobacco Sale at the POS

The law enforcement agents had never visited most vendors’ POS to ensure they complied with the tobacco control laws.

Interviewer: *Have the law enforcement agents come to your shop to see whether you comply with tobacco control laws?*

P9: *No, I’ve never seen them.*

Even when the law enforcement agents visited the vendors’ POS, they did not pay particular attention to cigarette sales. They were primarily interested in those who sold cannabis, which is banned in Nigeria.

##### Law Enforcement Agents Are Concerned about the Sales and Use of “jedi” (RYO)

The tobacco product that usually receives the agents’ attention is “jedi” (shredded tobacco used as RYO). However, the vendors’ account of how the enforcement agents deal with people who sell or use jedi was different. Some stated that the enforcement agents arrest those who sell or use “jedi”, similar to how they treat those who sell or use cannabis. Other vendors stated that because it looked like cannabis, the agents only checked to confirm it was jedi and would leave them without making an arrest once they confirmed that it was not cannabis.

However, most of the participants avoid selling “jedi” because it often attracts the attention of enforcement agents because of its semblance to cannabis.

P24: *I sell Time, Rothmans, Benson, Pall Mall, Esse; that’s what I sell. I do not sell jedi or rizlar [tobacco rolling paper] because I do not want a problem. … because the moment you start selling jedi and rizlar, you are closer to selling cannabis, and I don’t want anyone to arrest me.*

## 4. Discussion

The success, or otherwise of tobacco control laws regarding retail settings largely depends on the tobacco vendors’ compliance with the sections of the National Tobacco Control Act, 2015 (NTCA) relevant to their business. This study explored the vendors’ compliance with the tobacco-control laws related to the tobacco retail business. 

Most of the study participants were females, and even though there was an attempt to purposively select a more gender-balanced study population, it was difficult to achieve this. This suggests that females dominate the tobacco retail business in Ibadan, Nigeria, and probably across the country. This finding is supported by a quantitative study conducted in Oyo State, Nigeria, where the authors reported that 95% of the tobacco vendors were females [7]. Most studies on tobacco vendors do not pay attention to the sex of the vendors [16,17,18,19]. Sex has been associated with compliance with existing laws, with females being more willing to comply with the laws than males [20]. 

The three most common reasons the vendors stated for selling tobacco products were the pressure/demand from their alcohol-consuming customers, the need to earn profit for daily living, and the influence of their close family members. Tobacco and alcohol use are two strongly associated behaviors [13,21]. Those who drink alcohol are more likely than those who do not drink to smoke tobacco, and vice versa [13,21]. Hence, the demand for tobacco from someone who sells alcohol would be high and can be a motivation for them to combine sales of tobacco products with their alcohol sales. Similar to the finding in this study, a previous study in Pakistan also reported that the demand for tobacco products was a major reason the vendors commenced the tobacco business [22]. 

Some participants commenced tobacco sales due to the need to earn a living and the belief that selling cigarettes is a lucrative business. Thus, vendors should be discouraged from commencing tobacco sales by making it less lucrative and less profitable, and this can be done by increasing the tax on the products. However, the government should also support the vendors to engage in the sale of other non-tobacco products as a replacement for tobacco sales. Many of the vendors are likely to embrace this gesture, because, contrary to their initial belief before they commenced the tobacco business, most of the vendors reported that the profit from cigarette sales is very low and their overall profits from other non-tobacco products were higher. This study finding is similar to previous studies conducted in high-income countries (HICs) [23,24,25,26] and LMICs [27]. The result of this study contradicts the usual claims of the tobacco industries that the vendors are making a lot of profit from tobacco sales, and stricter laws by the government will cause serious economic losses for the vendors [26,28,29]. The tobacco industry claims that many vendors would be out of business, thus increasing unemployment [28]. However, several studies have shown that this claim by the tobacco industry is largely incorrect [29,30]. The participants in this study corroborated this stance by declaring that they would simply switch to non-tobacco alternatives and remain in business. Some were already considering stopping cigarette sales and focusing on other non-tobacco products.

The reason many of the vendors have continued selling cigarettes, despite the limited profit, was because they believed tobacco sales generate a ‘footfall’—an increase in the number of people/smokers visiting their stalls—who can potentially buy other non-tobacco products. This is one of the tobacco industry’s claims [25,26] and has been reported by other tobacco vendors [23]. However, there have been several arguments on footfall from tobacco sales in driving sales and profits from non-tobacco products. While the claims of the tobacco industry and the vendors have been largely subjective, empirical post-purchase surveys indicated that the footfall from selling tobacco products does not significantly contribute to sales and profits for non-tobacco products [24,31,32,33]. The vendors should be reassured that stopping tobacco sales and switching their focus to selling non-tobacco products is not likely to hurt their income. 

Awareness of laws is crucial for compliance, as vendors are less likely to comply with the law they never knew existed. Consistent with findings from a previous study in Oyo State, Nigeria [7] and other LMICs [34,35], most vendors in this study were unaware of Nigerian tobacco control laws affecting retail settings [7]. Thus, the relevant government agencies and other stakeholders must carry out public sensitization and educational campaigns about the tobacco control laws in Nigeria. If these educational programs are effectively executed, they will positively influence vendors’ compliance with the laws as it had been successfully used in other countries [36,37,38]. 

Considering the low level of awareness of the laws among the vendors in this study, the level of compliance was understandably, generally poor—a common finding in studies conducted in LMICs [6,34,39,40,41,42,43,44]. None of the participants in this study displayed the signage that tobacco cannot be sold to minors, and they all sold single sticks of cigarettes. Half of the participants had minors selling for them, and most of those who did not use minors were because they did not have access to children. Most participants in this study did not comply with the law banning sales to minors, which is a common practice in LMICs [34,43,44]. Although some participants claimed they do not sell tobacco to minors, further interaction showed that these vendors only meant they do not sell to minors who intend to use the product. However, they usually sell to children they believe were sent on errands by adults. Sending minors on errands to purchase tobacco products is common in Nigeria [45], and many vendors do not ask for the age of the buyers [46]. Instead, as this study has shown, the vendors usually decide the buyer’s age solely on their appearance, and this contravenes the law which stipulates that the vendor should “verify the age of the purchaser by checking any form of official identification” [12]. 

In contrast to the low compliance with the sale of tobacco to minors in this study, a study in Mumbai, India, reported that many retailers complied with the law [6]. However, the Mumbai study assessed self-reported compliance, and there were no indications that the vendors were asked specifically about selling to minors who were sent on errands. The findings in this study have exposed the possible error in self-reported compliance with the law banning tobacco sales to minors. Many vendors did not know that selling tobacco to minors running errands was illegal, so when they were asked if they complied with the ban, they simply answered “yes”, albeit incorrectly. Thus, studies, especially those utilizing self-administered questionnaires, should note and address this potential bias. 

The reason most of the vendors did not sell to minors whom they believed intended to use the products themselves was their perceived “social obligation” to protect the children from the negative health effects of tobacco use, and this is a similar finding in previous studies [22,47]. The perception of the participants about the different tobacco laws was directly related to their willingness to comply with the specific laws. Their perceptions of each law, after they were informed by the interviewer, could be classified into positive, negative, and indifferent. Most of the vendors had a positive perception of the ban on tobacco sales to and by minors. They considered it unhealthy for a child to use tobacco because of the increased risk of addiction and other harmful effects. This finding is similar to that of previous studies from HICs which reported that over 90% of the vendors had a positive perception of the ban on sales to minors [47,48]. Thus, it is unsurprising that they were all willing to comply with the laws banning tobacco sales to and by minors. These findings agree with the result of a previous study conducted in Oyo State, Nigeria, where the majority of the vendors were also willing to support the legislation on the ban of sales to minors and were willing to display signage [7]. 

Many of the vendors in this study were also willing to comply with the law banning tobacco advertisements and mandating that signage showing that tobacco sales to minors are not allowed. Again, this is likely due to their ‘indifferent’ perception towards the two laws. Many participants in this study claimed they usually do not advertise because the tobacco business does not need to be advertised, particularly since they were also selling alcohol, and potential customers know that when a vendor sells alcohol, they would also have tobacco on sale.

The implication is that the effective enforcement of the ban on tobacco advertisement and product display may not lead to the desired result if the same is not done for alcoholic drinks. Reports [49,50], have shown that the tobacco and alcohol companies have alliances to undermine policies and limit the impact of legislation on their businesses. They have various marketing strategies to jointly promote their products and reinforce brand appeals [49,50]. Hence, as Jiang and Ling (2011) opined, a holistic approach, including the effective ban on both tobacco and alcohol advertisement and product display, should be adopted as one of the tobacco control strategies in Nigeria and other countries with similar practices [50]. 

The vendors had an overwhelmingly negative perception of the law banning the sale of single cigarette sticks, and most were not willing to comply with the law. One of their reasons was that they did not want retail sales of tobacco products to be a capital-intensive business. With a little money, the vendors could buy a few packs of cigarettes, sell them as single sticks, and make a profit. With a law compelling them to sell only packs, they would need more capital to buy rolls and cartons of cigarettes, and they believed it would be difficult for them to get such huge capital. Some of the vendors also mentioned that the profit they make when they sell 20 single sticks of cigarettes is usually higher than when a pack of 20 sticks is sold at once. Many of the vendors were also against this law because they believed most of their customers would not be able to afford cigarette packs, and this could result in a reduction in demand for cigarettes. 

The vendors’ negative perception and unwillingness to comply with the ban on the sale of single sticks means that relevant agencies need to pay more attention to educating the vendors and enforcing the law. Studies [47,51,52], have shown that active enforcement of tobacco control laws, including frequent visits to the POS and effecting penalties for offenders, were positively associated with vendors’ compliance with the law. The vendors in this study stated that the enforcement agents were not educating nor enforcing the tobacco control laws. Although the possible reasons were not stated and beyond the scope of this study, we believe that it may be due to the enforcement agents’ lack of awareness of the laws or their roles in implementing them. We, therefore, recommend that the government and other stakeholders should ensure that all the relevant agencies are aware and equipped to carry out the implementation and enforcement of the tobacco control laws. 

This study is not without its limitations. Being a qualitative study conducted among purposively selected tobacco vendors in Ibadan, Nigeria; the result may not be generalizable to vendors in other settings. However, while the findings must be interpreted cautiously, the findings do not conflict with other studies.

## 5. Conclusions

This study showed that the overall level of compliance of the vendors to tobacco laws related to the retail business was very poor, and the major reasons for the lack of compliance were their lack of awareness of the laws and nonexistent enforcement. The participants expressed a positive view on the ban on sales to minors and sales by minors. They were indifferent to the ban on tobacco advertisement, product display, and displaying signages, but they all held a negative perception concerning the ban on sales of single cigarette sticks. 

Most vendors reported that while tobacco sales generate footfall, the profit is low, and the proportion of their overall profit from tobacco sales was minimal. Most of them did not foresee a serious economic effect on their overall business if they completely stopped selling tobacco. Overall, the participants were willing to comply with the laws.

## Figures and Tables

**Table 1:** Sociodemographic characteristics and tobacco use status of the study participants.

Variable	Frequency	Percent (%)
Age range (31–70 years)		
31–50 years	14	58.3%
51–70 years	10	41.7%
Duration as a vendor		
1–4 years	8	33.3%
5–9 years	6	25.0%
10–40 years	10	41.7%
Sex		
Male	4	16.7%
Female	20	83.3%
Tribe		
Yoruba	22	91.6%
Igbo	1	4.2%
Hausa	1	4.2%
Educational level		
No formal education	5	20.8%
Primary school	4	16.7%
Secondary school	12	50.0%
Tertiary school	3	12.5%
Study area		
Urban	16	66.7%
Semi-Urban	8	33.3%
Location of retail store		
Roadside/Street corner	8	33.3%
Mini-market	7	29.2%
Motor parks/Bus stops	9	37.5%
Type of POS		
Sales Counter	3	12.5%
Kiosk	4	16.7%
Shop	14	58.3%
Mini bar	3	12.5%
Tobacco products sold		
Cigarettes	24	100%
Roll-your-own (also called jedi)	5	20.8%
Vendors’ tobacco use status		
Current user	2	8.3%
Past user	1	4.2%
Never user	21	87.5%

**Table 2:** The themes and subthemes on tobacco vendors’ perceptions and compliance with tobacco control laws in Nigeria.

Themes	Subthemes	Sample of Representative Verbatim Quotes
Vendors’ reasons for commencing tobacco sales	Repeated demands from potential customers	P1*: When I first came here, I didn’t sell cigarettes. I sold beer and some other things. But people usually complain that I’m selling beer, but I don’t sell cigarettes. So those who wanted cigarettes would go to other shops [to buy cigarettes and beer]. Therefore, one day, I decided to start selling cigarettes too.*
	The hope of earning a profit and making a living	P10*: I started selling cigarettes because of my daily bread.*
	Influence from close family relatives	P12: *My new husband [who also smokes] told me to be selling cigarettes.*
Vendors’ awareness of the tobacco control laws	Lack of awareness of most tobacco laws related to the retail setting	*P2*4: *I have never heard of any laws.*
	Awareness about the law banning tobacco sales to minors	P14: *The laws are already written on it [pointed to the cigarette pack.], it is only for a person at age of 18 years and above*
Vendors’ perception of the laws	Positive perceptions of the laws that banned tobacco sales to and by minors	P14: *Yes, it will be good. If you want to do business, it will be better to find an adult that you will be paying a salary to assist you than to destroy your children’s future.*
	Indifference to the laws that banned tobacco advertisements and mandated the display of signage stating that minors are not allowed to buy tobacco	P19: *You do not need to advertise this market [cigarette]. I’m telling you the truth, it’s like MTN “everywhere you go”. Anywhere you keep it, you will sell it because many people are looking for it, so there is no need to advertise it. Anywhere you are, you will sell; even if you are selling it in your house, they will find you, not to talk of a public place like this.*
	Negative perceptions of the law that banned the sales of single sticks of cigarettes	P17: *We should keep selling single sticks because not all fingers are equal. Some people cannot afford a single stick sometimes; is it those people you will expect to buy a pack?*
Vendors’ compliance status with the laws	Not compliant	P1: *There is a child who helps me sell if I’m not around.*
	Compliant with some laws	P14: *I have a female child, and I don’t let her sell on my behalf. When she arrives from school, she goes home straight.*
Reasons for the participants’ compliance status	Lack of awareness of the laws	P2: *There are no laws regulating cigarette sales except for marijuana.*
	Knowledge of the health effects of tobacco on children	P13: *As a mother, I will not sell cigarettes to a child [who intends to smoke it]. So, based on the “die young” that is written on the packs. That is why I don’t like selling it to younger ones. But if it is an adult, I have no problem with that.*
	Focus on profit from sales	P3: *When you look at the age of the young boys who smoke cigarettes, you will see that they are about the age of 16 years. And at that age, they start smoking, which ought not to be. But we just overlook it because we bought it [cigarette] with money and we need to sell it and make our profit, so that’s why we sell it for them at times.*
Potential economic effect of stopping tobacco sales	Limited profit from tobacco sales	P9: *We can say that there is profit in it. However, it’s not something much, but it sells fast. Let’s say I buy six packs of cigarettes; it is possible to sell all six packs on the same day. If I gain ₦50 on each pack, this makes ₦300 [$0.72] in total.*
	The proportion of overall profit from tobacco sales	Interviewer: *Assuming you made a total profit of ₦100 in a day, how much of this profit is usually from cigarettes/tobacco sale?* P5: *I think it will be ₦20 or ₦30 [20%–30%]. That’s why I said the sales are fast, but not that the profit is much.*
	Vendors’ perception of the potential economic impact of ending tobacco sales	P5: *Honestly, it won’t have any effect. I said I don’t make much profit, so if people are asked to stop selling tobacco today, it will not have any effect on my overall business.*
Vendors’ experience with the activities of the enforcement agents	Lack of education and enforcement of the laws relating to the retail settings by the enforcement agents	P17: *Nobody has ever come here to educate me about the laws [tobacco control laws]*
	Enforcement agents’ activities regarding the roll-your-own tobacco product (jedi)	Interviewer: *Oh, does that mean that the police arrest people who sell “jedi”?* P12: *Yes, they arrest them [jedi smokers] a lot. They had met people smoking cigarettes in my shop before; they searched all their bodies down to their underpants, but they didn’t find anything like marijuana or jedi on them; that was when they released them.*

**Table 3:** The participants’ compliance with the tobacco control laws in Nigeria.

Specific Laws	Compliant	Frequency	Percent (%)
Ban on sales of single cigarettes stick	Yes	0	0.0%
	No	24	100.0%
Ban on sales to minors	Yes	6	25.0%
	No	18	75.0%
Ban on sales by minors	Yes	2	8.3%
	No	12	50.0%
	No access to a minor	10	41.7%
Signage stating tobacco sales to minors are prohibited	Yes	0	0.0%
	No	24	100.0%
Tobacco advertisement	Yes	20	83.3%
	No	4	16.7%
Tobacco promotion	Yes	16	66.7%
	No	8	33.3%
Product display	Yes	0	0.0%
	No	24	100.0%

## Data Availability

The data presented in this study are available on reasonable request from the corresponding author.

## Supplementary Materials

The following supporting information can be downloaded at: https://www.mdpi.com/article/10.3390/ijerph20227054/s1, Figure S1: Question guide; Table S1: Codebook - tobacco vendors’ compliance with the laws.
