# Supplementary figures and images for: Tobacco Vendors’ Perceptions and Compliance with Tobacco Control Laws in Nigeria
Source: Int J Environ Res Public Health. 2023 Nov 12;20(22):7054. doi: 10.3390/ijerph20227054 (PMC10671655; doi:10.3390/ijerph20227054)

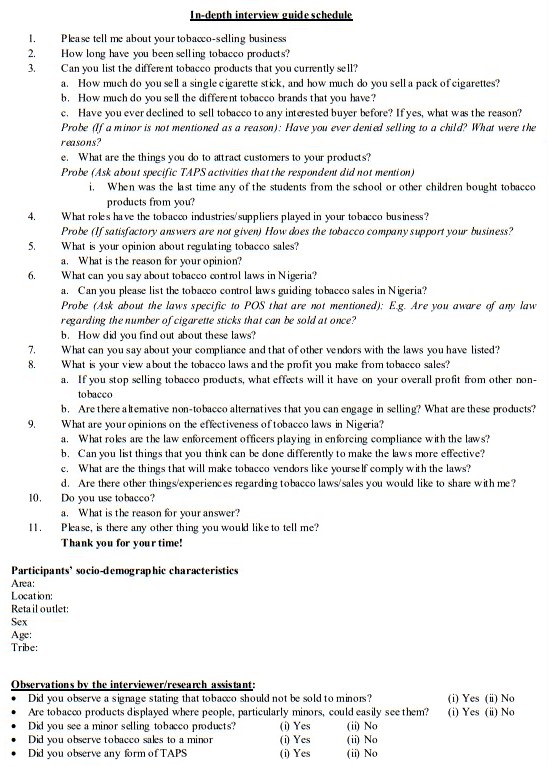

Supplement: Supplementary file 1 [file ijerph-20-07054-s001.zip › ijerph-2581548-Figure S1.JPG]
