# Supplementary material for: Tobacco Vendors’ Perceptions and Compliance with Tobacco Control Laws in Nigeria
Source: Int J Environ Res Public Health. 2023 Nov 12;20(22):7054. doi: 10.3390/ijerph20227054 (PMC10671655; doi:10.3390/ijerph20227054)
Supplement: Supplementary file 1 [file ijerph-20-07054-s001.zip › ijerph-2581548-Table S1.zip!/ijerph-2581548-Table S1.pdf]

CODEBOOK

Tobacco vendors' perceptions and compliance with tobacco control laws in Nigeria

Themes	Codes	Description/Definition	Sample of representative verbatim quotes
Vendors' reasons for commencing tobacco sales	Request from tobacco users	The participant (tobacco vendor) commenced the sales of tobacco because potential customers (tobacco users) complained about the lack of tobacco sales by the vendor.	***P1****: When I first came here, I didn't sell cigarettes. I sold beer and some other things. But people usually complain that I'm selling beer, but I don't sell cigarettes. So those who wanted cigarettes would go to other shops [to buy cigarettes and beer]. Therefore, one day, I decided to start selling cigarettes too.*
	Daily bread	The participant started the tobacco business to eke out a living	***P10:*** *I started selling cigarettes because of my daily bread.*
	Advice	The participant was advised to start tobacco sales	***P12****: My new husband [who also smokes] told me to be selling cigarettes.*
Vendors' awareness of the tobacco control laws	Unaware of the laws	The participant has never heard or seen anything about the tobacco control laws that relate to retail settings. Particularly, the ban on tobacco sales to and by minors, the ban on sales of single cigarette sticks, the ban on point of sale (POS) advertisements and product display, and the need to have signage stating that tobacco sales to minors are prohibited	***P24****: I have never heard of any laws.*
	Aware of the law	The participant can mention a law or respond affirmatively when prompted.	***P14:*** *The laws are already written on it [pointed to the cigarette pack.], it is only for a person at age of 18 years and above*
Vendors' perception of the laws	Support the law	The participant agrees that the law is good.	***P14:*** *Yes, it will be good. If you want to do business, it will be better to find an adult that you will be paying a salary to assist you than to destroy your children's future.*
	Neither in support nor against the law	The participant does not express a negative or positive response/reaction to the stated law.	***P19:*** *You do not need to advertise this market [cigarette]. I'm telling you the truth, it's like MTN "everywhere you go". Anywhere you keep it, you will sell it because many people are looking for it, so there is no need to advertise it. Anywhere you are, you will sell; even if you are selling it in your house, they will find you, not to talk of a public place like this.*
	Against the law	The tobacco vendor disagrees or express a negative response about the law	***P17:*** *We should keep selling single sticks because not all fingers are equal. Some people cannot afford a single stick sometimes; is it those people you will expect to buy a pack?*
Vendors' compliance status with the laws	Not compliant	The participant's conduct/activities contravene the laws, either based on their response to the questions or based on the interviewer's observations.	***P1:*** *There is a child who helps me sell if I'm not around.*
	Compliant	The participant's activities is in line with the stated laws.	***P14:*** *I have a female child, and I don't let her sell on my behalf. When she arrives from school, she goes home straight.*

Reasons for the participants' compliance status	Awareness of the law	The reason the participant is compliant or otherwise, with the law is related to their knowledge of the law.	***P2:*** *There are no laws regulating cigarette sales except for marijuana.*
	Health effects of tobacco use	The participants state that their concern with the health effects of tobacco use is a reason for their compliance with the law.	***P13:*** *As a mother, I will not sell cigarettes to a child [who intends to smoke it]. So, based on the "die young" that is written on the packs. That is why I don't like selling it to younger ones. But if it is an adult, I have no problem with that.*
	Profit	The potential financial gain from tobacco sales is a reason for their activities/compliance with the law.	***P3:*** *When you look at the age of the young boys who smoke cigarettes, you will see that they are about the age of 16 years. And at that age, they start smoking, which ought not to be. But we just overlook it because we bought it [cigarette] with money and we need to sell it and make our profit, so that's why we sell it for them at times.*
Potential economic effect of stopping tobacco sales	Profit margin from tobacco sales	Participants' description of how lucrative the tobacco business is: low, medium, high.	***P9:*** *We can say that there is profit in it. However, it's not something much, but it sells fast. Let's say I buy six packs of cigarettes; it is possible to sell all six packs on the same day. If I gain ₦50 on each pack, this makes ₦300 [$0.72] in total.*
	Proportion of overall profit	The participants' estimate of how much of the total profit from their business (tobacco and non-tobacco products) is from tobacco sales.	*Interviewer: Assuming you made a total profit of ₦100 in a day, how much of this profit is usually from cigarettes/tobacco sale?*<br>***P5:*** *I think it will be ₦20 or ₦30 [20% – 30%]. That's why I said the sales are fast, but not that the profit is much.*
	Potential Effect of stopping tobacco sales	The participants' response on the likelihood that their whole business will collapse if they are asked or "forced" to stop selling any tobacco product.	***P5:*** *Honestly, it won't have any effect. I said I don't make much profit, so if people are asked to stop selling tobacco today, it will not have any effect on my overall business.*
Vendors' experience with the activities of the enforcement agents	Education about tobacco laws	The participant's responses to the question asking if the enforcement agents or other relevant stakeholders had ever educated them about the law or enforced the law in their POS	***P17:*** *Nobody has ever come here to educate me about the laws [tobacco control laws]*<br><br>***Interviewer:*** *Have the law enforcement agents come to your shop to see whether you comply with tobacco control laws?*<br>***P9:*** *No, I've never seen them.*
	Confusion about jedi	The participants' description of the conflicting actions of the enforcement agents about the sales and use of jedi	***Interviewer****: Oh, does that mean that the police arrest people who sell "jedi"?*<br>***P12****: Yes, they arrest them [jedi smokers] a lot. They had met people smoking cigarettes in my shop before; they searched all their bodies down to their underpants, but they didn't find anything like marijuana or jedi on them; that was when they released them.*
